# A Study on Preparation and Stabilizing Mechanism of Hydrophobic Silica Nanofluids

**DOI:** 10.3390/ma11081385

**Published:** 2018-08-08

**Authors:** Mingwei Zhao, Wenjiao Lv, Yuyang Li, Caili Dai, Hongda Zhou, Xuguang Song, Yining Wu

**Affiliations:** Petroleum Engineering, State Key Laboratory of Heavy Oil Processing, China University of Petroleum (East China), Qingdao 266580, Shandong, China; lwj940502@163.com (W.L.); sunliyuyang@163.com (Y.L.); zhd940123@163.com (H.Z.); songxuguang1995@163.com (X.S.); wuyining@126.com (Y.W.)

**Keywords:** silica nanoparticle, nonionic surfactant, particle size, zeta potential, stability

## Abstract

Nanofluids have increasingly drawn interest in recent years with their various applications in a number of fields. The method for the preparation of stable nanofluids is a key concern for extending the application of nanofluids. This study focuses on the effect of pH, dosage of surfactant (TX-100), and nanofluid concentration on the stability of a silica nanofluid. Particle size and zeta potential are two important factors to consider in evaluating the stability of the silica nanofluid. Results indicate that the stability of the silica nanofluid highly depends on pH, dosage of surfactant (TX-100), and nanofluid concentration. On the basis of these experiments, the best conditions for the preparation of a silica nanofluid are 0.1 wt. % for the concentration of silica nanoparticles and TX-100 and 10 for pH. A transparent and stable silica nanofluid can thus be obtained.

## 1. Introduction

The suspension of solid particles in liquids is widely known to contribute to industrial liquid systems, such as heat transfer fluids, lubricant fluids, and magnetic fluids [1,2,3,4,5]. Among these technologies, nanofluids as engineering materials consisting of nanometer-sized additives and base fluids have attracted interest because of their broad applications in heat transfer, cooling of microchips, drug delivery, and enhanced oil recovery [6,7,8,9,10,11,12].

Preparation of nanofluids is the first step to experimental studies and industrial use. Two general methods for the preparation of nanofluids have been identified: the single-step method and the two-step method. The single-step method involves the simultaneous preparation of nanoparticles and nanofluids. Liu [13] et al. synthesized Cu nanofluids by adding Cu nanoparticles in water by chemical reduction. Zhu [14] et al. presented a single-step preparation of Cu nanofluids by the reduction of CuSO_4_·5H_2_O with NaH_2_PO_2_·H_2_O in ethylene glycol under microwave irradiation. Meanwhile, the two-step method involves the dispersion of synthesized nanopowders into a fluid by using different mechanical, physical, and chemical techniques. Using the two-step method, Xie [15] et al. prepared Al_2_O_3_/EG and Al_2_O_3_/PO nanofluids, and Murshed [16] et al. prepared TiO_2_–water nanofluids.

Regardless of the method employed to prepare nanofluids, nanoparticles tend to aggregate over time because of their high surface activity, thus limiting their application. In most studies, various adjustments have been applied to improve the stability of nanofluids—changing the pH value, adding dispersants, modifying the surface of the nanoparticle, and using ultrasonic agitation. Numerous studies proved that modifying the pH value, dosage of dispersants, and concentration of nanofluids induces a change in the stability of nanofluids, affecting their properties (thermal conductivity, viscosity, and so on) [17,18,19,20,21,22]. Therefore, the stability of nanofluids should be improved, and the factors influencing this stability should be investigated.

Hwang [23] et al. dispersed carbon black in water and silver in silicon oil and examined the stability of nanofluids; the nanofluids prepared using the two-step method exhibited good stability for about 60 d. Peng [24] et al. identified nanoparticle concentration, dispersant, viscosity of base liquid, and pH as the most important factors affecting the stability of nanofluids. Li [25] et al. demonstrated that pH, dispersant type, and concentration influenced the stability of Cu–water nanofluids; the addition of optimizing dispersant concentrations at pH of 9.5 can also lead to the best stability of Cu–water nanofluids.

Silica nanoparticles are widely used because of their satisfactory abrasion resistance, electrical insulation, and environmental protection [26]. Many studies have been conducted on the preparation of silica nanofluids [27,28,29,30,31]. Li et al. [30] prepared silica water-based nanofluids composed of synthesized silica nanoparticles and evaluated their ability for enhanced oil recovery. We [31] also synthesized benzimidazole-modified silica nanoparticles and dispersed them into brine to obtain silica nanofluids. However, the preparation of stable silica nanofluids by using a simple method remains a great challenge.

Studies have been conducted on the addition of ionic surfactants (SDS, CTAB, and so on) [23,32,33,34] to improve the stability of nanofluids. Owing to their relative sensitivity to salinity, surfactants are further limited in application. In the present study, we designed a two-step method to prepare silica nanofluids by mixing silica nanoparticles and Triton X-100 (TX-100), a nonionic surfactant. The effects of pH, surfactant, and nanoparticle concentrations on the dispersion of silica nanofluids were evaluated using effective diameters and zeta potential. The influence rule of nanofluids was concluded based on the results.

## 2. Experimental Section

### 2.1. Materials

Hydrophobic silica nanoparticles (Aladdin Reagents Company, Shanghai, China) were used in this study. The nanoparticles had a specific surface area of 380 ± 30 m^2^/g and an average diameter of 8 nm. NaOH (AR), HCl (AR), and TX-100 (CP) were purchased from Xilong Chemical Company, Shantou, China. Deionized water was used in all experiments.

### 2.2. Preparation of Nanofluid

Silica nanoparticles were first mixed vigorously with TX-100 in water. Subsequently, 1 mol/L NaOH and 1 mol/L HCl were used to modulate the pH level. Mechanical stirring and ultrasonic dispersion effectively dispersed highly entangled or aggregated nanoparticles. The mixture was stirred for 10 min and the nanoparticles were evenly dispersed using a KQ-300 DE ultrasonic dispersion instrument (Kunshan Ultrasonic Instruments Company, Kunshan, China) at a frequency of 40 kHz and an output power of 100 W at 25 °C. Silica nanofluids were obtained after 1 h.

### 2.3. Characterization

The effective diameters and zeta potentials of the silica nanofluid under different conditions (concentration of nanofluid and surfactant, pH) were measured using the NanoBrook Omni laser particle size analyzer (Brookhaven Instruments Company, Holtsville, NY, USA).

## 3. Results and Discussion

### 3.1. The Influence of pH on Stability of Silica Nanofluid

**Figure 1:**
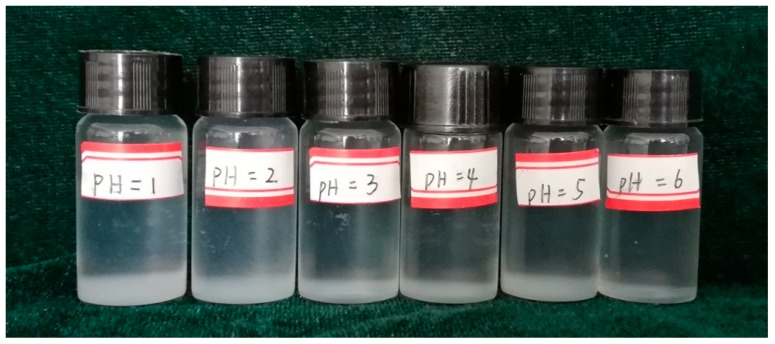
Silica nanofluid under different acid conditions (Image Taken after adding HCl for 1 h).

The pH level is an important factor affecting the stability of nanofluids. In this study, a surfactant (TX-100) was used as a dispersing agent to obtain transparent and stable nanofluids. Silica nanoparticles (0.1 g) were mixed with TX-100 (0.1 g) in water to prepare a 100 mL nanofluid. After the suspensions were stirred thoroughly and then ultrasonicated for at least 1 h, NaOH and HCl were used to modulate the pH. According to the results, with a pH of 1–7, the silica nanofluid could not remain transparent after adding HCl for several minutes and its stability was interrupted (Figure 1). As a typical nonionic surfactant, TX-100 could be adsorbed on the surface of silica nanoparticles and thus form a protective film to allow silica nanoparticles to be dispersed in water, which is referred to as a steric hindrance effect. When HCl was added, hydrogen ions were adsorbed and neutralized on the silica surface, thereby reducing the stability of the silica nanofluid. Therefore, a transparent and stable nanofluid could not be obtained under acidic conditions.

**Figure 2:**
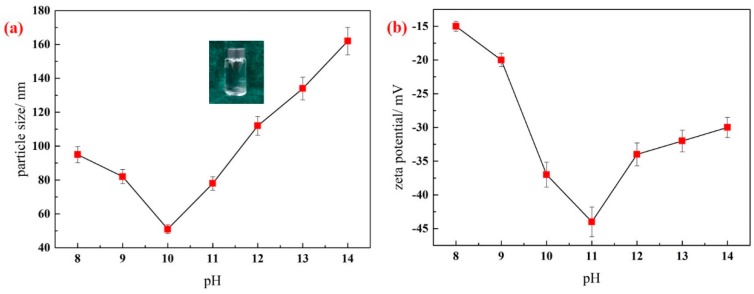
Effect of pH value on the particle size (**a**) and zeta potential (**b**) of silica nanofluid. Error bar = RSD (relative standard deviation) (*n* = 5).

With a pH of 8 to 14, the silica nanofluid remained transparent. The zeta potential and particle size were measured to evaluate the effect of pH on the stability of silica nanofluids. Each experiment was repeated at least five times to calculate the mean of the experimental data. Figure 2 shows that with an increase in NaOH, the hydraulic particle size and zeta potential of the silica nanofluid changes. The curve in Figure 2b exhibits a trend consistent with that in Figure 2a. As pH increases from 8 to 10, the particle size of the silica nanofluid decreases from 95 nm to 51 nm. As pH continues to increase, the particle size of the silica nanofluid increases to 160 nm. The particle size indicates the stability of the silica nanofluid: when the particle size is small, the silica nanoparticles have a low probability of aggregation. Figure 2b shows that the absolute values of zeta potential change greatly from 15 mV to 45 mV when pH increases from 8 to 11. Despite a decrease with a continuous increase in pH, the absolute values of zeta potential are not lower than 30 mV. The zeta potential of ±30 mV is regarded as a critical value for evaluating the stability of the nanofluids. A lower zeta potential (<30 mV) leads to particle agglomeration, whereas a higher zeta potential (>30 mV) can maintain stability) [35], which indicates that the silica nanofluid can remain stable with an increase in pH.

The study of the effect of pH on the stability of nanofluids shows that the zeta potential of nanoparticles plays an important role in the stability of the nanofluid. Changing the pH value is an effective method to modulate the zeta potential of nanoparticles. With a larger zeta potential, high electrical repulsion between nanoparticles can be generated, limiting the aggregation of the particles. With a lower zeta potential, electrical repulsion between nanoparticles is generated, which cannot sufficiently prevent the aggregation of nanoparticles, leading to the low stability of nanofluids. The key point in nanofluid stability is the modulation of the zeta potential of nanoparticles. As shown in Figure 2, when pH is from 8 to 10, the absolute value of zeta potential increases as pH increases. The electrostatic repulsion between the particles is sufficient to prevent the aggregation of particles. A larger electrostatic repulsion also renders the particles relatively independent, which indicates that the dispersion stability of the silica nanofluid is improved, and the particle size decreases. When pH increases to 11–14, the dosage of NaOH increases, thereby decreasing the electrostatic repulsion and increasing the particle size. When pH is about 10, the particle size of the silica nanofluid is smallest, the absolute values of zeta potential are high, and the best dispersion and stability of the nanofluid system are obtained.

### 3.2. The Influence of Surfactant Dosage on Stability of Silica Nanofluid

**Figure 3:**
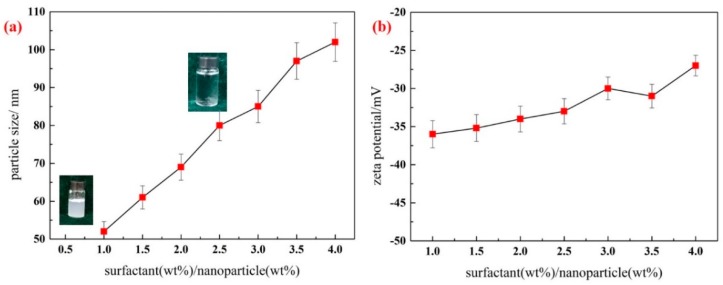
Effect of dosage of TX-100 on particle size (**a**) and zeta potential (**b**) of silica nanofluid (pH = 10). Error bar = RSD (*n* = 5).

As determined in the aforementioned experiments, a pH of 10 is an appropriate condition for the preparation of the silica nanofluid in this study. To evaluate the effect of surfactant concentration on the stability of the silica nanofluid, 0.1 wt. % silica nanoparticles were mixed with TX-100 at different concentrations in water to prepare silica nanofluids. The resulting zeta potentials and particle sizes are shown in Figure 3.

**Figure 4:**
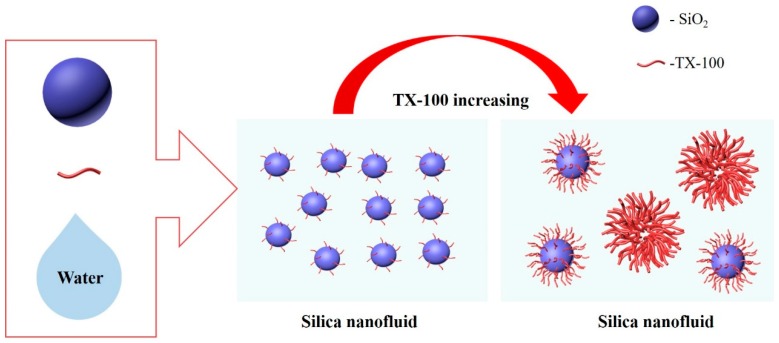
Effect of dosage of TX-100 on stability of silica nanofluid (pH = 10).

When TX-100 has a low dosage (surfactant/silica nanoparticles = 0.5, mass ratio), the protective film on the surface of the silica nanoparticle is weak and cannot provide sufficient repulsive force to render silica nanoparticles stable, resulting in an opaque silica nanofluid. As shown in Figure 3a, the particle size of the silica nanofluid increases rapidly with an increase in TX-100 dosage, which indicates that the silica nanofluid becomes unstable. TX-100 as a dispersant is a nonionic surfactant. When the concentration is higher than the critical micelle concentration (cmc), micelles can be formed in the solution. In the nanofluid, after TX-100 is added into the solution, TX-100 molecules can be adsorbed on the solution surface, form micelles, and be adsorbed on the surface of silica nanoparticles. In the present study, silica nanoparticles are hydrophobic. The dispersant should be dispersed to prepare stable nanofluids. After TX-100 is adsorbed on the surface of silica nanoparticles, the hydrophobic hydrocarbon chain can be entangled on the surface of silica nanoparticles, forming a protecting layer. Hydrophilic headgroups of TX-100 are observed on the outer surface, as shown in Figure 4. When TX-100 concentrations increase, more TX-100 molecules are adsorbed on the surface of silica nanoparticles. Owing to steric hindrance, TX-100 molecules are more loosely arranged, resulting in an increase in the hydraulic radius of silica nanoparticles. In addition, as the TX-100 dosage continues to increase, a double-layer adsorption is observed on the surface of silica nanoparticles. Some hydrophobic groups extend into water, causing silica nanoparticles to become unstable in water.

As shown in Figure 3b, no substantial change in the zeta potential of the silica nanofluid occurs with an increase in TX-100 dosage. TX-100 as a nonionic surfactant cannot be ionized in water and exerts reduced influence on the surface charge of silica nanoparticles. This experiment leads to the conclusion that surfactant concentration considerably affects the stability of silica nanofluids; when the mass ratio of surfactant to silica nanoparticle is 1, the smallest particle size and large absolute values of zeta potential of silica nanofluids are obtained. The nanofluid system exhibits the best dispersion and stability.

### 3.3. The Influence of Nanoparticle Concentration on Stability of Silica Nanofluid

**Figure 5:**
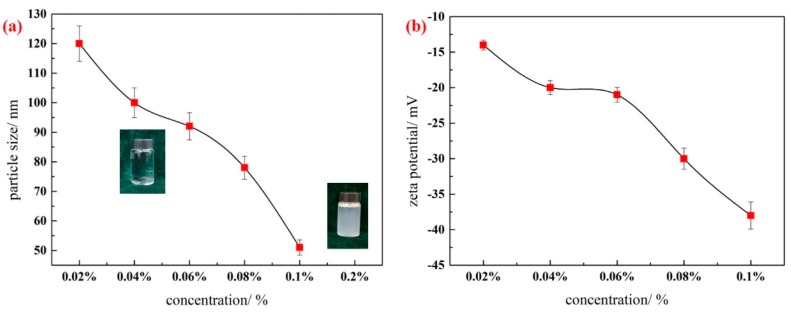
Effect of concentration of nanofluid on particle size (**a**) and zeta potential (**b**) of silica nanofluid (pH = 10, surfactant/nanoparticles = 1). Error bar = RSD (*n* = 5).

To evaluate the effect of nanoparticle concentration on the stability of the silica nanofluid, a series of silica nanofluids with different concentrations (0.02 wt. %, 0.04 wt. %, 0.06 wt. %, 0.08 wt. %, 0.1 wt. %, and 0.2 wt. %) were prepared. The mass ratio of the surfactant to silica nanoparticles was set to 1, and the pH levels of all nanofluids were adjusted to 10. The results indicate that stable silica nanofluids with different concentrations can be prepared, except that with a concentration of 0.2 wt. %. According to Figure 5, the trend of changes in particle size is perfectly consistent with that of the potential zeta values. This finding agrees with the aforementioned experimental results. If the zeta potential value is larger, causing increased electrostatic repulsions, the hydraulic radius decreases. With an increase in silica nanoparticle concentrations, the particle size of the silica nanofluid decreases, and the absolute values of zeta potential increases, indicating that the stability of silica nanofluids is enhanced with an increase in concentration. The preparation conditions are thus determined to be 0.1 wt. % for the silica nanoparticle concentration and TX-100 and 10 for the pH value.

### 3.4. The Stability Mechanism of Silica Nanofluid

Both the pH value and dispersant concentrations are regarded as important factors influencing the stability of the silica nanofluid, as determined by the aforementioned experiments [33,36,37]. The pH value changes the zeta potential of the silica nanofluid, thereby changing the electrostatic repulsion between nanoparticles. When pH is lower than 10, the particle charge and thickness of the TX-100 adsorption layer are reduced, consequently decreasing the space electrostatic repulsion and increasing the free surfactant molecules. The particle size and stability of the silica nanofluid decrease.

**Figure 6:**
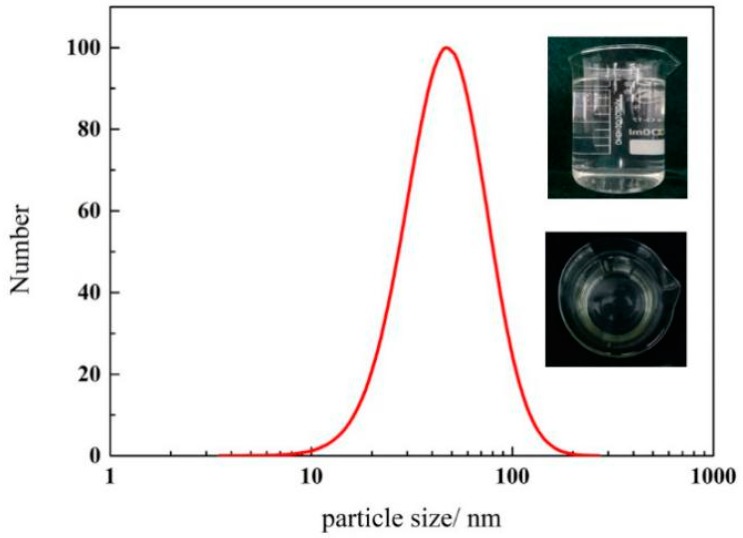
Particle size distributions of silica nanofluid.

**Figure 7:**
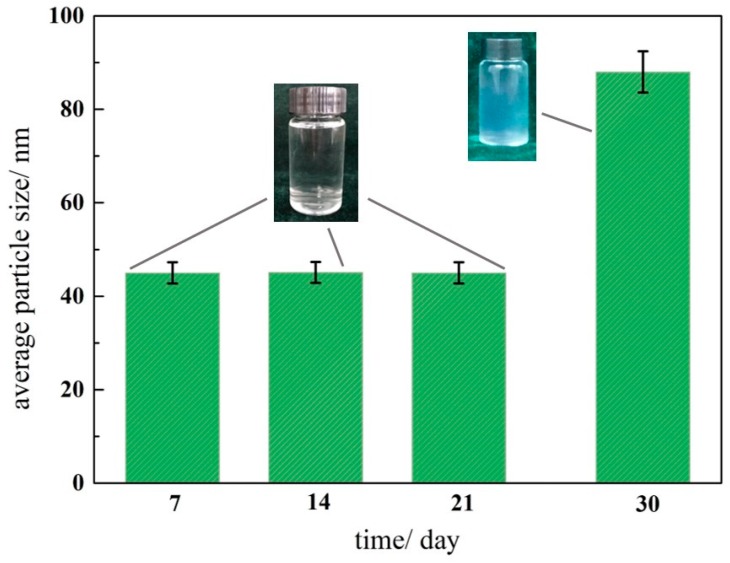
Average size of silica nanofluid at different reserve time. Error bar = RSD (*n* = 5).

Surfactant dosage also affects the adsorption layer. When the surfactant dose is higher, double-layer adsorption occurs, and TX-100 micelles can be generated, resulting in the agglomeration of silica nanoparticles. Therefore, appropriate pH and dosage are important for the preparation of silica nanofluids. A transparent and stable silica nanofluid with the concentration of the silica nanoparticle and TX-100 equal to 0.1 wt. % and pH of is 10 was prepared. Figure 6 shows the particle size distributions of the silica nanofluid. The average particle size of this dispersion is about 45 nm, with a size distribution of 10–100 nm, and the zeta potential is about −35 mV. In the laboratory, the prepared silica nanofluid can remain stable for three weeks at room temperature (Figure 7). The silica nanofluid exhibits good stability, which ensures its applicability.

## 4. Conclusions

This study focused on the preparation of stable silica nanofluids. The particle size and zeta potential were measured to evaluate the stability of the nanofluid under different conditions. Key conclusions were drawn:

(1) A silica nanofluid was prepared using the two-step method. TX-100 was used as the dispersant to allow the dispersion of hydrophobic silica nanoparticles in water.
(2) The pH value markedly influenced the silica nanofluid. Silica nanoparticles could not be dispersed uniformly under acidic conditions. The pH value of 10 was considered optimal.
(3) Optimal TX-100 dosage and concentration were determined. According to the results, the silica nanofluid exhibited the highest stability when the surfactant-to- silica nanoparticle ratio was 1 and the nanofluid concentration was 0.1 wt. %.

## Nomenclature

*ζ*	zeta potential/mV
*c*	concentration/%
*d*	particle size/nm
*D*	reserve time/day
*RSD*	relative standard deviation
